# Manual of Physiology

**Published:** 1853-05

**Authors:** 


Art. XIII.—*Manual of Physiology,* by William Senhouse Kirkes, m. d., Licentiate of the Royal College of Physicians; Registrar and Demonstrator of Morbid Anatomy, at St. Bartholomew's Hospital. Assisted by James Paget, f. r. s., Lecturer on General Anatomy and Physiology at St. Bartholomew's Hospital. Second American from the second London edition, with 165 illustrations. Philadelphia, Blanchard & Lea, 1853. 12mo pp. 568.

In some respects, the physiology of Kirkes & Paget is unrivalled in the English language. It will not compare with Carpenter's Principles of Human Physiology in fullness of detail, but it is not inferior to that admirable work in the qualities of clearness, accuracy, good arrangement, and fidelity to the facts of the science, while its brevity is a recommendation as a text-book for students. We have already more than once borne testimony to the excellence of Dr. Carpenter's physiological works, and we could not say anything that would add to their popularity; but the great size to which his 'Human Phisiology' has grown renders it somewhat unwieldy. It is becoming unmanageable as a manual, and as such must give place to the less ambitious, but hardly less complete treatise of Kirkes and Paget. In this treatise, physiology is presented in its most modern guise, and with sufficient detail to make every subject intelligible, and to give to the work all the interest that will be found in larger treatises.

The publishers of Carpenter have done well to issue this "Manual;" the two works do not interfere with each other, and the practitioner as well as the student of medicine may read both with advantage. The facts of physiological science possess the highest interest for the physician, and we are glad that works in which they are placed in so captivating a light are now accessible to every member of the profession. Of the practical bearing of physiology upon medicine let us, in concluding this brief notice, give a single illustration, afforded by recent researches into the constitution of human urine.

It has been understood that the urine is variable as to its reaction; at one time acid, at another alkaline; that it is affected by disease and by diet; and remedies have often been addressed with a view to the correction of what was deemed a constant condition of the fluid. Recently it has been discovered that the condition of the urine is a most variable one, and that its acidity is inversely as that of the stomach, decreasing for a time after taking food, reaching the minimum from three to five hours after a meal, and frequently giving place to an alkaline reaction. Its maximum acidity is after a fast of some hours, when the condition of the stomach is neutral. If the individual continue to fast, the state of the urine remains stationary for ten or twelve hours. The stomach being habitually acid keeps the urine in an alkaline condition and favors urinary concretions. The acid reaction of the urine is due to the presence of acid phosphate of soda.

From this example we see that if the urine were examined at one hour, an inference would be drawn as to therapeutics the opposite to that which a subsequent investigation would authorise.

